# The association between hemogram parameters and the development of contrast-induced nephropathy in patients presenting with non-ST-elevation myocardial infarction

**DOI:** 10.1515/almed-2023-0037

**Published:** 2023-08-14

**Authors:** Esra Dönmez, Sevgi Özcan, İrfan Şahin, Ertuğrul Okuyan

**Affiliations:** Department of Cardiology, Bağcılar Training and Research Hospital, Bağcılar, İstanbul, Türkiye

**Keywords:** CIN, contrast induced nephropathy, mean platelet volume, neutrophil lymphocyte ratio

## Abstract

**Objectives:**

Hemogram parameters such as mean platelet volume (MPV), neutrophil/lymphocyte ratio (NLr), red cell distribution width and platelet distribution width are widely used inflammatory indicators to assess prognosis in various cardiovascular diseases. In this study, we aimed to investigate the role of hemogram parameters to predict the development of contrast-induced nephropathy (CIN) in patients presenting with non-ST segment elevation myocardial infarction (non-STEMI) and treated with percutaneous coronary intervention (PCI).

**Methods:**

All pateints who underwent PCI with a diagnosis of non-STEMI between 2017 and 2020 in our center were included retrospectively in this study.

**Results:**

A total of 387 patients were included in this retrospective study. Advanced age (p=0.001, β:0.005, OR [95 % CI]: 0.002–0.007), diabetes mellitus (p=0.013, β:0.205, OR [95 % CI]: 0.150–0.260), congestive heart failure (p=0.009, β:0.095, OR [95 % CI]: 0.024–0.166), volume of contrast medium (p=0.008, β:0.241, OR [95 % CI]: 0.184–0.392), MPV (p=0.02, β:0.047, OR [95 % CI]: 0.028–0.065) and NLr (p=0.001, β:0.052, OR [95 % CI]: 0.040–0.063) were found as independent risk factors associated with CIN development according to multivariate logistic regression analysis. A cut off value of 5.5 for NLr was associated with 79.6 % sensitivity and 79.5 % specificity and 9.05 for MPV was associated with 64.1 % sensitivity and 58.7 % specificity in prediction of CIN development.

**Conclusions:**

Hematological parameters, assessed by routine blood count analysis may serve as a promising and useful marker for CIN especially when used in combination with traditional risk factors. MPV and NLr were demonstrated as predictors of CIN development in non-STEMI patients who were treated with PCI in our study.

## Introduction

Contrast-induced nephropathy (CIN) is a kind of acute kidney injury associated with intravascular contrast media (CM) use and one of the well-known complications of cardiac catheterization procedures. CIN is defined as the increase in serum creatinine (Cr) level at least 0.5 mg/dL or 25 % than baseline serum Cr within 48–72 h after contrast medium exposure [1]. Its incidence is approximately 2–5 % in general population however, patient [comorbidities such as advanced age, chronic renal disease (CRD), diabetes mellitus (DM), congestive heart failure (CHF)], procedure (acute coronary syndrome, complex coronary interventions) related factors and amount of CM may cause higher prevalence (up to 20–30 %) [2]. Since CIN development is known to be associated with in-hospital and long-term mortality and morbidity, early diagnosis and attempts to define patients under risk may alter progression and prognosis [3, 4].

Vasoconstriction, direct cellular toxicity and inflammation are risk factors for CIN development [5], [6], [7]. Hemogram parameters such as mean platelet volume (MPV), neutrophil/lymphocyte ratio (NLr), eosinophil, red cell distribution width (RDW) and platelet distribution width (PDW) are widely used inflammatory indicators to assess prognosis in various cardiovascular diseases [8], [9], [10], [11]. Studies examining the role of hemogram parameters in development of CIN are limited. In this study, we aimed to investigate the role of hemogram parameters to predict CIN development in patients presenting with non-ST segment elevation myocardial infarction (non-STEMI) and treated with percutaneous coronary intervention (PCI).

## Materials and methods

All patients who underwent PCI with a diagnosis of non-STEMI between 2017 and 2020 in our center were included retrospectively in this study. Local hospital electronic database and patients’ files were screened to obtain demographic, clinical and laboratory parameters. Medical treatments which were either applied in hospital or have been previously in use by the patients were also recorded. Non-ST elevation myocardial infarction was diagnosed according to recent guidelines [12]. Presence of stenosis ≥50 % in two or more epicardial coronary arteries are defined as multivessel disease. Patients with a history of leukemia, thrombocytopenia, end-stage renal disease, malignancy, chronic autoimmune disease and/or patients under steroid or nonsteroidal anti-inflammatory therapy and contrast medium exposure within the last 2 weeks were excluded. Patients with a decision of medical treatment, multivessel PCI or emergent surgery based on coronary angiography were also excluded. Physiological (0.9 %) saline given intravenously at a rate of 1 mL/kg/h and 0.5 mL/kg/h those with reduced ejection fraction (EF) or CHF for 12 h after contrast exposure was applied as a routine follow-up treatment in our clinic. A non-ionic, low-osmolality contrast agent was used in our catheterization laboratory.

Congestive heart failure diagnosis was based on a previous history of heart failure, or objective evidence of reduced left ventricular ejection fraction (LVEF) ≤40 % assessed by echocardiography on admission [13]. Transthoracic echocardiography was performed in all patients (Vivid S70; GE Medical System, Horten, Norway) and left ventricular ejection fraction (LVEF) was measured using Simpson’s method. Hypertension (HT) was defined as prescribed medications for lowering blood pressure, any measurement of over 140/90 mmHg prior to operation and/or a previous formal diagnosis [14]. Stroke was defined as any history of neurological deficits lasting more than 24 h that resulted from impaired cerebral blood flow [15]. Reversible neurological dysfunction causing symptoms less than 24 h was defined as transient ischemic attack (TIA) [16]. A fasting blood glucose level of ≥126 mg/dL (7.0 mmol/L) and/or hemoglobin A_1c_ value>6.5 % or use of antidiabetic medicine was indicative of diabetes mellitus (DM) [17]. Coronary artery disease (CAD) was defined as 50 % luminal diameter stenosis in at least one major epicardial vessel by diagnostic coronary angiography.

Hemogram parameters (hemoglobin, RDW, PDW, MPV, platelet count, neutrophil, monocyte and eosinophil counts) which were obtained at first admission recorded (Cell Dyn 3700; Abbott Diagnostics, Lake Forest, Illinois, USA). Baseline serum Cr level obtained from first admission biochemical analysis was accepted as ‘baseline Cr’ and the maximum serum Cr level which was obtained at least 48 h after contrast administration was accepted as ‘maximal Cr’ value. Two groups generated according to CIN development; CIN developed as CIN (+) and CIN non-developed as CIN (−). The primary endpoint of this study was the occurrence of CIN. Human Studies and Research Committee of our institution approved the study and patient consent was waived accordingly.

### Statistical analysis

All statistical tests were conducted using the Statistical Package for the Social Sciences 22.0 (SPSS Inc., Chicago, IL, USA). Continuous variables are expressed as mean ± SD, and categorical data are expressed as number (n) and percentages (%). Chi-square test was used to assess differences in categorical variables between groups. Student’s t-test or Mann Whitney U test was used to compare unpaired samples as needed. Variables having linear correlation were evaluated by using Pearson’s correlation test and nonlinear variables were evaluated by using Spearman’s correlation test. The correlation of hemogram parameters with CIN development was investigated. Logistic regression analysis was used to identify independent variables of CIN in the study population. Area under the receiver operating curve (ROC) was calculated to assess the ability of hemogram parameters to estimate outcomes. Significance was assumed at a 2-sided p<0.05.

## Results

Five hundred and twenty-nine patients were evaluated, after exclusion of patients as defined in methodology and those with lack of data, finally a total of 387 patients were included in this retrospective study. The mean age was 65.9 ± 10.9 and 60.5 % were male. Baseline Cr level was 1.05 ± 0.28. When patients were grouped according to CIN development as CIN (+) and CIN (−); 74 (19.1 %) patients formed the CIN (+) and 313 (80.9 %) patients formed CIN (−) group. Both groups were similar in terms of gender, body mass index, smoking status, hyperlipidemia, HT, history of stroke and received medical treatment. However, advanced age (70.4 ± 9.2 vs. 64.8 ± 11.1; p<0.0001), DM (66.2 % vs. 26.2 %; p<0.0001), patients with a history of CAD (64.8 % vs. 32.3 %; p<0.0001), CHF (40.5 % vs. 10.5 %; p<0.0001) and volume of CM used (142 ± 39 vs. 101 ± 34; p=0.004) were significantly higher in CIN (+) group. In terms of laboratory markers; maximal urea (78.2 ± 46.4 vs. 46.7 ± 19.3; p<0.0001), maximal Cr (1.69 ± 0.74 vs. 1.04 ± 0.29; p<0.0001), uric acid (7.1 ± 1.9 vs. 5.9 ± 2.2; p=0.003), troponin (40.6 [4.3–5,000] vs. 32.0 [1–3,590]; p=0.002), fasting blood glucose (169.9 ± 85.4 vs. 140.1 ± 70.2; p=0.002) were significantly higher and albumin (3.8 ± 0.5 vs. 4.2 ± 0.7; p=0.002) level was significantly lower in CIN (+) group. Furthermore, PDW (16.2 ± 4.3 vs. 14.8 ± 2.7; p=0.016), MPV (9.4 ± 2.5 vs. 8.2 ± 1.3; p=0.035) and NLr (5.3 ± 1.8 vs. 3.9 ± 1.1; p<0.0001) were significantly higher in CIN (+) group. All demographical, clinical and laboratory characteristics of the two groups are presented in detail in Table 1.

**Table 1:** Clinical and demographic data of study population and two groups according to contrast induced nephropathy development.

Variables	All n=387	Group-1 CIN (+) n=74	Group-2 CIN (−) n=313	p-Value
**Clinical characteristics and comorbidities**				

Age, years	65.9 ± 10.9	70.4 ± 9.2	64.8 ± 11.1	**<0.0001**
Male, n (%)	234 (60.5)	44 (59.5)	190 (60.7)	0.844
BMI, kg/m^2^	28.7 ± 4.4	28.5 ± 4.4	28.7 ± 4.4	0.673
Smoker, n (%)	102 (26.5)	18 (24.3)	84 (27.0)	0.638
HT, n (%)	224 (57.9)	45 (60.8)	179 (57.2)	0.068
HPL, n (%)	42 (10.9)	9 (12.2)	33 (10.5)	0.687
DM, n (%)	131 (33.9)	49 (66.2)	82 (26.2)	**<0.0001**
Previous CAD, n (%)	149 (38.5)	48 (64.8)	101 (32.3)	**<0.0001**
Previous stroke, n (%)	11 (2.8)	2 (2.7)	9 (2.9)	0.586
Previous CHF, n (%)	63 (16.3)	30 (40.5)	33 (10.5)	**<0.0001**
LVEF, %	48.2 ± 11.2	46.4 ± 11.6	48.6 ± 11.1	0.176
Volume of CM, mL	119 ± 62	142 ± 39	101 ± 34	**0.004**
Drugs, n (%)				
Aspirin	292 (75.4)	58 (78.4)	234 (74.8)	0.136
ACE inhibitor/ARB use	219 (56.5)	44 (59.5)	175 (55.9)	0.084
Calcium channel blocker	101 (26.1)	17 (22.9)	84 (26.8)	0.097
β-Blocker	127 (32.8)	24 (32.4)	103 (32.9)	0.584

**Laboratory parameters**				

Urea, mg/dL				
Baseline	45.1 ± 19.5	47.7 ± 19.6	44.5 ± 19.4	0.200
After 48 h	52.6 ± 29.3	78.2 ± 46.4	46.7 ± 19.3	**<0.0001**
Creatinine, mg/dL				
Baseline	1.05 ± 0.28	1.11 ± 0.29	1.03 ± 0.28	0.059
After 48 h	1.16 ± 0.49	1.69 ± 0.74	1.04 ± 0.29	**<0.0001**
Hemoglobin, g/dL	13.2 ± 1.8	12.8 ± 1.9	13.1 ± 1.9	0.385
Hematocrit, %	39.4 ±°5.3	39.1 ± 5.3	39.4 ± 5.2	0.691
PDW, %	15.7 ± 3.9	16.2 ± 4.3	14.8 ± 2.7	**0.016**
RDW, %	14.1 ±°1.5	13.9 ± 1.5	14.1 ± 1.5	0.705
MPV, fL	8.7 ± 1.4	9.4 ± 2.5	8.2 ± 1.3	**0.035**
WBC × 10³/μL	8.47 ±°4.92	8.67 ± 5.76	7.89 ± 4.39	0.087
Platelet counts 10³/μL	223 (147–494)	219 (147–481)	217 (149–494)	0.258
NLr	3.7 ± 2.1	5.3 ± 1.8	3.9 ± 1.1	**<0.0001**
Albumin, g/dL	4.3 ± 0.6	3.8 ± 0.5	4.2 ± 0.7	**0.002**
Uric acid, mg/dL	6.2 ± 2.2	7.1 ± 1.9	5.9 ± 2.2	**0.003**
ProBNP, pg/mL	77.8 (50–28,679)	96.5 (186–28,679)	76.1 (50–26,528)	0.439
Troponin T, pg/mL	39.0 (1–5,000)	40.6 (4.3–5,000)	32.0 (1–3,590)	**0.002**
Fasting glucose, mg/dL	145.9 ± 74.2	169.9 ± 85.4	140.1 ± 70.2	**0.002**
AST, U/L	26.0 (8–1,245)	31.0 (8–1,245)	25.0 (10–1,105)	0.079
ALT, U/L	23.0 (5–540)	21.0 (9–540)	25.0 (5–540)	0.101
Total cholesterol, mg/dL	184.1 ± 52.2	182.6 ± 43.3	184.5 ± 52.9	0.782
Triglycerides, mg/dL	170.9 ± 112.5	147.4 ± 57.2	176.4 ± 121.3	0.052
LDL cholesterol, mg/dL	111.3 ± 39.3	111.9 ± 35.4	111.2 ± 40.2	0.878
HDL cholesterol, mg/dL	41.7 ± 11.9	41.5 ± 9.4	41.8 ± 12.5	0.873

ACE/ARB, angiotensin converting enzyme/angiotensin receptor blocker; ALT, alanine aminotransferase; AST, aspartate aminotransferase; BMI, body mass index; BNP, brain natriuretic peptide; CAD, coronary artery disease; CHF, congestive heart failure; CIN, contrast induced nephropathy; CM, contrast medium; DM, diabetes mellitus; HDL, high density lipoprotein; HPL, hyperlipidemia; HT, hypertension; LDL, low density lipoprotein; LVEF, left ventricular ejection fraction; MPV, mean platelet volume; NLr, neutrophil lymphocyte ratio; PAD, peripheral artery disease; PDW, platelet distribution width; RDW, red cell distribution width; WBC, white blood cell. p<0.05 was accepted as statistically significant.

**Figure 1:**
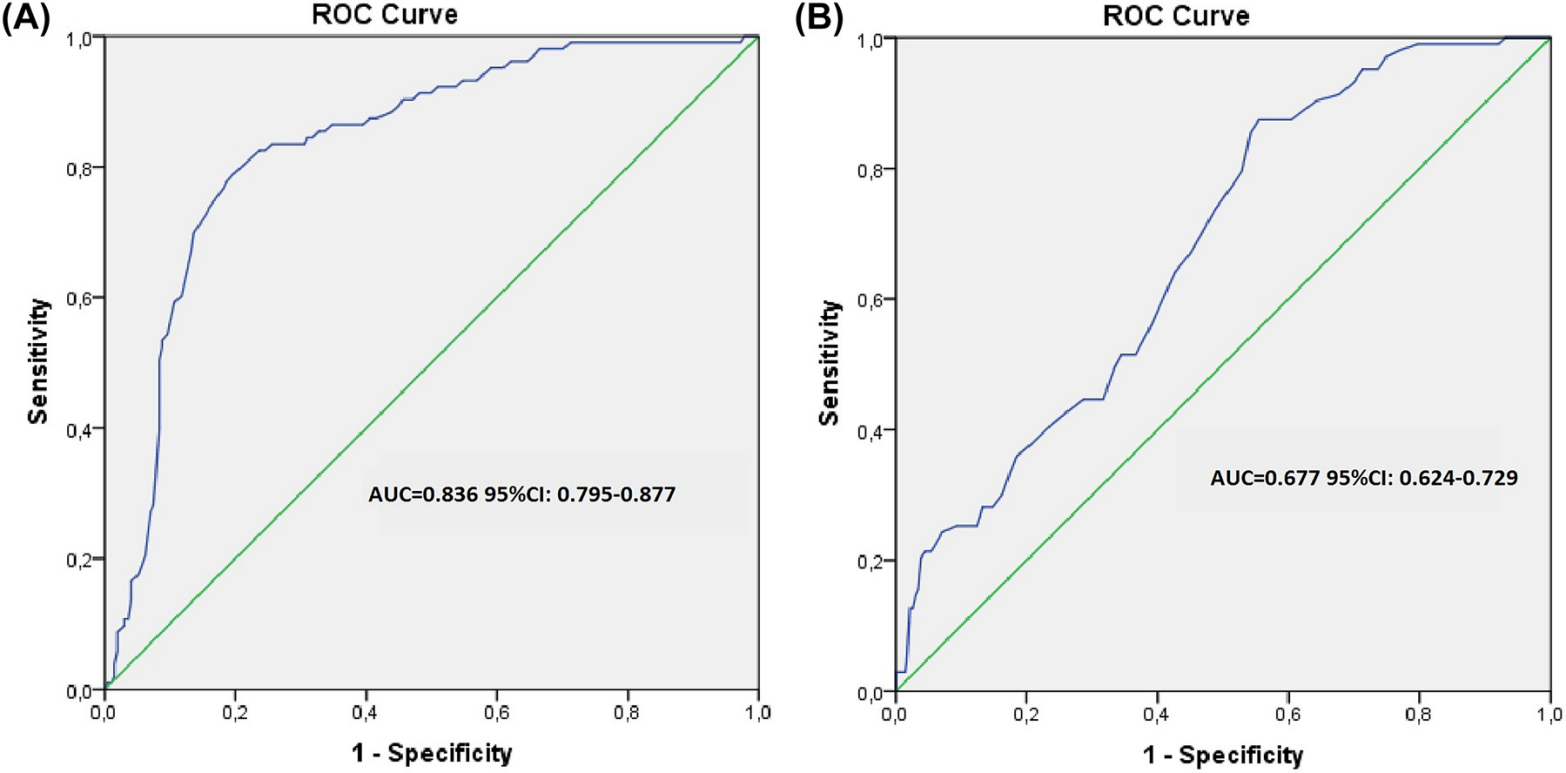
ROC curve for accuracy of hemogram parameters for predicting CIN development in non-STEMI patients. (A) Area under the ROC curve of neutrophils/lymphocytes ratio and development of CIN in patients with non-STEMI. (B) Area under the ROC curve of mean platelet volume and development of CIN in patients with non-STEMI.

To further evaluate individual risk factors for CIN development, univariate logistic regression analysis was performed for age, DM, history of CAD and CHF, volume of CM, albumin, uric acid, troponin, fasting glucose, PDW, MPV levels and NLr, respectively. By univariate logistic regression analysis, advanced age, volume of CM used, history of DM, CHF, albumin, PDW, MPV levels and NLr were found to be correlated with CIN development. These variables were assessed in the multivariate logistic regression model. Advanced age (p=0.001, β: 0.005, OR [95 % CI]: 0.002–0.007), history of DM (p=0.013, β: 0.205, OR [95 % CI]: 0.150–0.260), history of CHF (p=0.009, β: 0.095, OR [95 % CI]: 0.024–0.166), volume of CM (p=0.008, β: 0.241, OR [95 % CI]: 0.184–0.392), MPV (p=0.02, β: 0.047, OR [95 % CI]: 0.028–0.065) and NLr (p=0.001, β: 0.052, OR [95 % CI]: 0.040–0.063) were found as independent risk factors associated with CIN development by multivariate logistic regression analysis (Table 2). ROC analysis was performed to identify the optimal cut-off value and area under the curve (AUC) for NLr. ROC for accuracy of NLr for predicting CIN development in non-STEMI patients is shown in Figure 1A. The AUC for NLr was 0.836 [%95 CI: 0.795–0.877]. A cut off value of 5.5 for NLr was associated with 79.6 % sensitivity and 79.5 % specificity in prediction of CIN development. Moreover, ROC curve for accuracy of MPV for predicting CIN development in non-STEMI patients is shown in Figure 1B. The AUC for MPV was 0.677 [%95 CI: 0.624–0.729]. A cut off value of 9.05 for MPV was associated with 64.1 % sensitivity and 58.7 % specificity in prediction of CIN development.

**Table 2:** Univariate and multivariate forward stepwise logistic regression analysis: predictors of CIN development.

	Univariate OR	95 % CI	p-Value	Multivariate OR	95 % CI	p-Value
Age	0.009	0.006–0.012	**<0.0001**	0.005	0.002–0.007	**0.001**
Diabetes mellitus	0.330	0270–0.391	**<0.0001**	0.205	0.150–0.260	**0.013**
Previous CAD	0.339	0.343–1.456	0.127			
Previous CHF	0.420	0.345–0.494	**0.001**	0.095	0.024–0.166	**0.009**
Volume of CM	0.268	0.093–0.444	**0.003**	0.241	0.184–0.392	**0.008**
Albumin	0.122	0.049–0.049	**0.030**	0.098	0.064–1.045	0.726
Uric acid	0.175	0.005–1.035	0.138			
Troponin T	0.174	0.008–1.158	0.881			
Fasting glucose	0.251	0.102–1.481	0.958			
PDW	0.259	0.114–0.389	**0.020**	0.814	0.189–1.002	0.113
MPV	0.067	0.046–0.088	**0.002**	0.047	0.028–0.065	**0.020**
NLr	0.050	0.034–0.067	**<0.0001**	0.052	0.040–0.063	**0.001**

CAD, coronary artery disease; CHF, congestive heart failure; CIN, contrast induced nephropathy; CM, contrast medium; MPV, mean platelet volume; NLr, neutrophil lymphocyte ratio; PDW, platelet distribution width. p<0.05 was accepted as statistically significant.

## Discussion

The prevalence of CIN development was found 19.1 % and hematological parameters were found to be useful for predicting CIN development in our study. Preprocedural assessment of the hematological parameters may raise suspicion to foresee the incidence of CIN in patients with non-STEMI. The independent predictors of CIN development were advanced age, history of DM, CHF, volume of CM, MPV and NLr. Close follow-up of patients with MPV>9.05 and NLr>5.5 on admission as an additional clue to other risk factors may help to define patients under risk of CIN development.

Contrast medium is a part of diagnostic and therapeutic approaches especially in cardiology era and CM use raised due to developments in interventional cardiology. CIN is one of the vital complications that can occur due to CM exposure. In addition to relation between CIN and increased morbidity/mortality, the prolonged hospital and intensive care unit stays and increased need for hemodialysis, CIN development may cause raised cost on health care system [18]. Inflammation, thrombosis, vasoconstriction, direct cellular toxicity and vascular remodeling are part of CIN pathophysiology. The impact of renin–angiotensin–aldosterone system blocking agents on CIN development is still controversial [19], [20], [21]. Advanced age, DM, CHF, HT, renal dysfunction, amount and type of CM, exposure to nephrotoxic agents, being in dehydrated status and urgent interventions are risk factors for CIN development. According to our results advanced age, DM, history of CHF and total CM volume were established as independent risk factors for CIN development. Although, history of CAD was observed significantly higher in CIN developed group; it was not found as independent predictor. Any effect of angiotensin converting enzyme inhibitor/angiotensin receptor blocker (ACEI/ARB) treatment on the incidence of CIN development was not observed in our study.

High RDW is a finding of impaired erythropoiesis and indicates raised oxidative stress and chronic inflammation. The prognostic role of high RDW in various cardiac circumstances such as CAD, acute coronary syndrome, CHF and paroxysmal atrial fibrillation was shown [9, 22]. The linkage between high RDW and CIN development was evaluated in patients who were treated with PCI with a diagnosis of chronic coronary syndrome and RDW was shown to have low predictive value in CIN development [23]. Distinct from chronic coronary syndromes, RDW was found to be a significant predictor of CIN development in patients presenting with ST-segment elevation myocardial infarction [24]. However, only patients presenting with NSTEMI were included and there was no relationship between RDW and CIN development according to our results. NLr was detected as a predictor of prognosis and major events in myocardial infarction and moreover an indicator of inflammation and proteinuria in patients with chronic kidney disease [25, 26]. NLr was established as an independent predictor of CIN development, attributable to the role of inflammation in the pathophysiology of CIN in our study. Mean platelet volume is an indicator of platelet size and activity reflecting the risk of plaque burden, plaque morphology, progression and vulnerability. Mean platelet volume was shown as an independent risk factor for cardiovascular diseases [27]. Larger platelets have increased thrombogenic properties. Platelet distribution width reveals the platelets which have different sizes with different metabolic and thrombogenic activity in circulation [28]. Although MPV and PDW were significantly higher in the group that developed CIN, only MPV was detected as independent predictor of CIN development in our study.

### Limitations

Our study was conducted in a single center and designed retrospectively. Evaluating additional inflammation markers such as high-sensitive C-reactive protein might give further information about the inflammatory status and opportunity to correlate with hematological parameters. Post-procedural creatinine level was obtained at least 48 h after contrast exposure; therefore, some patients who had a later increase in serum Cr levels after discharge may have been missed.

## Conclusions

Contrast medium use is increasing due to developments in diagnostic and therapeutic approaches in interventional cardiology. Contrast-induced renal injury leads to increased morbidity, mortality, health care costs and prolonged hospital stays. Iatrogenic and predictable nature of CIN marks it as a cornerstone for ongoing cardiovascular and nephrology research, with a focus on risk factors and preventative, diagnostic and therapeutic measures. A chronic inflammatory response may play a role in the pathogenesis of CIN, and hematological parameters, assessed by routine blood count analysis may serve as a promising and useful marker for CIN development especially when used in combination with traditional risk factors. Mean platelet volume and NLr were demonstrated as predictors of CIN development in non-STEMI patients who were treated with PCI in our study. Larger and prospective studies are needed to evaluate the relationship between hematological parameters and prevalence of CIN.
